# What Really Motivates Iranian Nurses to Be Creative in Clinical Settings?: A Qualitative Study

**DOI:** 10.5539/gjhs.v7n5p132

**Published:** 2015-02-24

**Authors:** Sara Shahsavari Isfahani, Mohammad Ali Hosseini, Masoud Fallahi Khoshknab, Hamid Peyrovi, Hamid Reza Khanke

**Affiliations:** 1 Nursing Department, University of Social Welfare and Rehabilitation Sciences, Tehran, Iran; 2 Department of Critical Care Nursing, School of Nursing and Midwifery, Iran University of Medical Sciences, Tehran, Iran

**Keywords:** creativity, innovation, motivation, nursing, qualitative content analysis, Iran

## Abstract

**Background:**

Creativity and innovation are key elements for organization improvement, particularly in nursing, and for finding alternatives for solving nurses’ occupational problems. Nurses’ creativity is affected by motivation. Although, there are many possible sources of motivation, the Iranian nurses’ creativity is seldom clarified, and the most important factors motivating nurses to be creative in clinical settings has rarely been addressed.

**Objectives:**

The aim of this study was to explore Iranian nurses’ experiences regarding the most important factors that motivate their creativity in clinical settings.

**Methods:**

This qualitative study was conducted using conventional content analysis approach. A purposive sample of sixteen nurses was recruited from two educational hospitals affiliated to Tehran and Jahrom Universities of Medical Sciences in Iran. Data were gathered through conducting face-to-face semi-structured interviews and were analyzed by qualitative content analysis approach.

**Findings:**

Five main themes emerged from the data analysis, including: (a) Intrinsic motivators, (b) Extrinsic motivators, (c) Achievement motivators, (d) Relational or altruistic motivators, and (e) Spiritual motivators.

**Conclusions:**

Study findings indicated that nurses are creative and innovative individuals. So nurse managers and health policy makers should consider creativity as an integral part of all health and clinical strategies and policies. They should support creative and innovative efforts of nurses and provide a climate in which nurses engage in more creative and productive behaviors.

## 1. Introduction

The world of healthcare is changing, so development of personnel performance is important and is the core of high quality healthcare (Toode, Routasalo, & Suominen, 2011). As the complexity of health care and nursing increases, health care providers are being challenged to think more creatively and develop innovations that advance the knowledge, learning, and services to the health care organizations (Pesut, 2013). Nursing is progressively becoming a diverse profession. The nurse in the 21st century will have to be a creative and innovative to cope effectively with new and unique elements in complex health care settings (Potgieter, 1999) and to find alternatives for solving problems related to the profession as a whole (Feldman, Ruthes, & Cunha, 2008). Effective nursing practice in the new millennium also will require innovative and creative nurses who can adapt to change and have the courage to take risks in order to provide holistic, individualized, and context-specific care (Potgieter, 1999). Creativity is not a new concept to the nursing profession. Nurses often encounter with unexpected situations and they take care of patients with different backgrounds and health conditions, hence they need to go beyond nursing routines and acquire creative thinking to make useful decisions (Chan, 2013). In most health care systems, nurses are the main professional member of ‘front line’ health care team providing up to 80% of primary health care. As such, they are critically responsible to provide creative and innovative solutions for the current and future global health challenges (Hughes, 2006).

Creativity is a mental activity and the most important performance dimension that is triggered by specific problems and results in novel solutions (Swansburg, 1996). The terms creativity and innovation are often used interchangeably, although, many researchers have suggested that they are two distinct areas (e.g., Amabile, 1996; Baron & Tang, 2011; Hopkins, 2010; McLean, 2005). Creativity is defined as the production of novel and useful ideas in any domain, while innovation is the successful implementation of creative ideas within an organization (Amabile, 1996). Creative ideas are starting points of all innovations. Baron and Tang (2011) also argue that creativity is a necessary condition for subsequent innovations.

Creativity is a function of three components: expertise, creative-thinking skills, and motivation (Amabile, 1998). Motivation is a value-based, psycho-biologically stimulus-driven inner urge that activates and guides human behavior in response to self, others, and environment, supporting intrinsic satisfaction and leading to the intentional fulfilment of basic human drives, perceived needs, and desired goals (Moody & Pesut, 2006). Nurses’ job motivation has been proven to be important for their intent to work (Brewer, Kovner, Greene, & Cheng, 2009). It provides direction and purpose in nurses’ work, however motivational factors for creative activities may be vary during different career stages and in different clinical settings. Amabile and Steven (2011) argued that many possible sources of motivation such as extrinsic, intrinsic, and relational or altruistic motivation exists that stand out as the most relevant to work life, though research about nurses’ creativity and what really are the most important motivational factors that affect Iranian nurses to be creative in clinical settings is very scarce.

Recent nursing literatures has also repeatedly proclaimed the need for creativity in nursing science (Potgieter, 1999), yet creativity has not been extensively studied in nursing practice and the factors that motivate nurses’ creativity at workplace is still not clear. Studies have also indicated that creativity is not highly rewarded in practice; however it is supposed to be in the theory (Sternberg, 2006a). The place of creativity within nursing education also appears underdeveloped and disconnected from health care settings (Schmid, 2005) and motivational factors that triggers nurses’ creativity in these settings are still unknown. Moreover, the most nursing creativity research in Iran have been conducted with quantitative approach (e.g., Jafarpour, Erfannia, & Rashid, 2011; Moshirabadi, Seyedfatemi, Borimnejad, Haghani, & Yazdanipour, 2013; Jokari, Jorfi, & Ebadi, 2012). Therefore considering existing evidence in Iran as well as lack of comprehensive qualitative studies in the area of creativity in nursing, this study was conducted to explore experiences of Iranian nurses regarding the most important factors that motivate creativity in clinical settings in the cultural context of Iran.

## 2. Method

### 2.1 Design and Aim

This qualitative study was conducted using the conventional content analysis approach. The aim of this study was to explore Iranian nurses’ experiences regarding the most important factors that motivate them to express creativity in clinical settings.

### 2.2 Setting and Participants

In this study, 16 nurses (female=6 and male=10) who had worked in different units of educational hospitals affiliated to Jahrom and Tehran Universities of Medical sciences in Iran were recruited by purposeful sampling with the maximum variation sampling to achieve variation in nurse’s gender and work experiences as well as educational levels. Two nurses refused to participate in the study after initial agreement (both because of problems in setting time for interview). Inclusion criteria for nurses consist of having novel and useful ideas, products, services or procedures related to nursing work, producing one or several different objective and creative products as part of their work in clinical settings and being interested to participate in the study. In this study, matron, supervisor, and head nurses were asked to introduce those nurses who expressed creativity and innovation at the workplace.

### 2.3 Data Collection

Data collection was conducted through a semi-structured interview. It was composed of two open-ended questions (interview guide) as follows: Have you had any experience regarding creativity in your workplace? And what factors make you to generate new ideas, products, services or procedures or to do creative efforts in the clinical setting? Also some probing questions were asked for additional clarification of the answers given by participants. No prejudices or personal opinions were involved in the interview process, and semi-structured guidelines were adopted to guide interviewees to express their experiences as far as possible. Data were collected from June 2012 to December 2013. Interviews lasted between 30 and 90 minutes and were performed in a quiet place in participants’ working units. After recording the interviews, they were transcribed verbatim immediately and analyzed at the same time. All interviews were performed by the first author and were audiotaped with the nurses’ consent. The interviewer was well experienced in qualitative curriculum training and had clinical practicum teaching experience which would help the participant to enter the interview situation and build a trustworthy relationship. The data collection, data analysis and participants’ selection were continued until data saturation occurred and a rich description of nurses’ experiences was obtained.

### 2.4 Data Analysis

Data analysis was performed using conventional content analysis approach. Content analysis consists of advanced techniques for data processing. It is a systematic approach which aims at providing a novel insight and a better understanding about phenomena under study and helps to identify their practical applications (Krippendorff, 2012). For analyzing the data, ‘Framework’ was used as a method of qualitative data analysis. ‘Framework’ is an analytical process which involves a number of distinct though highly interconnected stages (Bryman & Burgess, 2002). This method has five key stages. In the first stage or familiarization stage, the data transcribed verbatim and each interview was read several times to gain a sense of content. In the second stage or identifying a thematic framework, the text was divided into meaning units. The condensed meaning units were abstracted and labeled with a code, which constitute the manifest content. In the third stage or indexing, we compared the various codes based on differences and similarities and sorted them into sub-categories and categories and collated all the relevant coded into data extracts within the identified categories. In the fourth stage or charting, we read all the collated extracts for each category and considered whether they appeared to form a coherent pattern. Then, the validity of individual categories in relation to the dataset and whether our candidate categories “accurately” reflected the meaning evident in the dataset as a whole was considered. Two researchers independently examined the data for categories. The final stage or mapping and interpretation involves defining and then refining categories.

### 2.5 Ethical Considerations

The Ethics Committee of University of Social Welfare and Rehabilitation Sciences in Tehran approved this study (2ID11767). Before starting each interview, all participants were informed about the objectives and method of the study. They were also informed that participation in the study is voluntary; therefore they could refuse to participate or withdraw from the study at any time. Moreover, the participants were reassured that their responses would be kept confidential and their identities would not be revealed in research reports and publications of the study. Finally, the participants who agreed to participate in the study were asked to sign a written consent.

### 2.6 Trustworthiness

Trustworthiness is among the most important issues in qualitative studies and facilitates the evaluation of researchers’ effects and actions (Holloway, 2005). Confirmability, credibility, dependability and transferability were employed to assure various aspects of trustworthiness according to Lincoln and Guba (1985). For confirmability, the bracketing process was put aside assumptions and biases that were possessed by the researchers before data collection. To assure credibility, we used the maximum variation of sampling, peer debriefing or reviewing of the data, codes and themes by a co-researcher, and member checking of the findings by our participants. Focusing on the research objectives and trying to explore the same areas for all the participants were done by researchers during the study to assure dependability. Recruiting participants with the maximum variation of sampling and thick description of the findings helped transferability of the results.

## 3. Findings

In total, sixteen nurses (male=10 and female=6) between 27 and 57 years old participated in this study. Their work experiences were from 7 to 30 years and most of them worked in different units of the hospitals (Table 1). Five major categories emerged from the data analysis, including: (a) Intrinsic motivators, (b) Extrinsic motivators, (c) Achievement motivators, (d) Relational or altruistic motivators, and (v) Spiritual motivators. The rest of the text discusses the meaning of each theme, with quotations from participants.

**Table 1:** Participants’ characteristics

Variable	Number (%)
Age Distribution (Year)	
25-34	9(56.25%)
35-44 45-54 55-64	4(25%) 2(12.5%) 1(6.25%)
Gender	
Male	10(62.5%)
Female	6(37.5%)
Educational Level
MSN*	3(18.75%)
BSN**	10(62.5%)
ADN***	1(6.25%)
LPN****	2(12.5%)
Work Experience(Year)
5-9	3(18.75%)
10-14	7(43.75%)
15-19	3(18.75%)
20-24	1(6.25%)
25-29	1(6.25%)
30-34	1(6.25%)
Total	16

* Master of Science in Nursing;

** Bachelor of Science in Nursing;

*** Associate Degree in Nursing;

**** Licensed Practical Nurse.

### 3.1 Intrinsic Motivators

This was mentioned by all creative nurses who were interviewed. All Participants expressed intrinsic motivation in the form of broad interest to nursing profession or to love the nursing and interest to perform clinical practices. They mentioned “to love what you do” is one of the most significant factors and added that when a nurse is interested in his/her own works; he/she would express creativity spontaneously. Creative nurses stated that to love and to be interested in nursing encouraged them to make creative efforts and they felt satisfaction and happiness, especially when their new ideas are fulfilled and are used in the hospitals.

*“I think the most important factor was my interest to my job. If you love your work, you would pursue which work is better to do. In my opinion, loving what you do is the most important factor. I was interested in nursing and always seeking to do a bigger and better work for the patients and for the community” [nurse 1, 30 years work experience]*.

Creative nurses stated that one of the most prominent motivational factors that encouraged them to do creative efforts were intrinsic passion and interest in nursing.

*“Hospital officials and colleagues did not believe me. They told I am not able to do this. But because I was interested to my work and I loved it, I tried to make this special table (C-Arm table) to prove myself to them that “I can do” [nurse 2, 13 years work experience with one patent]*.

Participants recognized that love to nursing practices and serving the people is central to nursing. They were committed to nursing profession and mentioned one of the factors that cherish nursing profession is performing the nursing practices in regarding with love. Participants cited that love to work means “not to retreat” it means “to move forward”, and it results in self-actualization and authority. They stated that an internal sense propels them forward and they felt internal satisfaction when they performed creative efforts.

*“Although I knew that it (a Robot for pulling the patient’s legs during the orthopedic surgery) might never be made or patented at all, but I liked and I was interested to do it, I myself did not know too, an inner sense propels me forward and encouraged me” [nurse 3, 7 years work experience with 11 patents]*.

### 3.2 Extrinsic Motivators

Creative nurses stated that being present at the patients’ bedside and performing clinical practices, repeatedly confronted them with many clinical problems including patients and their co-workers problems. The participants reported that while they were carrying out nursing procedures, personal problems of their own creative nurses, hospital financial difficulties, sanctions, work overload, time pressure, shortage of nursing workforce and hospital equipments, and educational problems of patients in clinical settings were faced them with serious mental challenges. When they confronted with aforementioned clinical problems they carefully paid attention to and thought about them and seek to find several diverse alternatives in order to solve the problems and this in turn led them new ideas sparks into their minds. Because nurses faced with time pressure in the hospitals, more new ideas that they offered were in direction to made new devices that reduced the time and simplifies the works as works were perform faster, easier and more convenient, so that they could utmost use of their time and energy. Some of the nurses cited that real-workplace problems have the potential to increase motivation by allowing them to feel their learning is more relevant and meaningful to their own work lives.

*“I have ten national and one international patent that all of them were due to working in the patient bedside. These ideas sparked to my mind while I was working at the hospital” [nurse 3, 7 years work experience with 11 patents]*.

*“In the hospital when only one nurse does change dressing, I observed that his hands become contaminated when he touches the bottle of normal saline or betadine and this led to non-sterile change dressing. In that moment, this question came to my mind, is it possible to invent a device that automatically shed these solutions? Confronting with and careful observation of this problem resulted to sparks the idea of inventing automatic wash dressing machine into my mind” [nurse 3, 7 years work experience with 11 patents]*.

Participants also mentioned that support and encouragement of managers and colleagues and having used their new ideas in the hospital were the important motivators for them that offered new ideas. One nurse also stated that being rebuked and ridiculed by colleagues and head nurses because performing nursing procedures very slowly and disruption in other tasks in the ward were effective motivators in spark of the ideas. Participants mentioned that material incentives were less important for them than other motivators. Some of them stated that they do creative efforts because they like to gain social admiration, recognition and to earn reputation as well as to become popular. Only few of nurses stated that monetary incentives, obtaining high profits and income and achieving financial self- sufficiency were motivating factors.

### 3.3 Achievement Motivators

Creative nurses stated that engaging in creative efforts needs willingness to work hard and for long hours. It requires that they spend a lot of time and energy. They mentioned their enthusiasm helped them to create new ideas or to follow them and to bear these hard workings. Creative nurses felt that they need to change without any prejudice prior to change. Participants cited that they were interested in breaking old frames and they challenged the traditional ways of doing nursing procedures. Also, they liked to make progress and felt averse to routine nursing practices. They had developed their capabilities, knowledge, expertise and technical skills and integrated their technical and professional skills with their nursing knowledge. Creative nurses stated that being strong was necessary to prove their new ideas to the hospital and university officials and when they faced with their opposition, they were not disappointed or did not give up their efforts. Some of the participants also stated that they do creative efforts because they like to be the first and the most excellent employee in the workplace.

*“I said to myself; ‘well, I can do’, and then ‘why I limit the cardiovascular nursing educations in one hospital? I can do a bigger work’. And then, the idea of establishing of Cardiovascular Nursing Association sparked in my mind. However, the previous experiences also helped me to do it” [nurse 4, a founder of nursing association]*.

*“After hospital shifts, I went to a workshop to work with turnery and I was there till late. I made a robot that is able to pull the patient’s legs for the purpose of orthopedic surgery. I spend a lot of time, energy and money for it. But by no means, I did not feel exhausted. Because I liked to make it” [nurse 3, 7 years work experiences with 11 patents]*.

*“Indeed, I always find myself in the workplace that routine nursing procedures not motivated me. Most of the times, I tried to follow the issues that emerged for me in clinical practice and try to find the new problems and new ways to solve them” [nurse 5, 23 years work experience with one patent]*.

### 3.4 Relational or Altruistic Motivators

Creative nurses stated that they had careful and sophisticated attention to clinical setting problems. They stated that deeply understanding of the clinical status and perceiving the problems of the patients and their family members, colleagues, doctors, clinical students and other personnel resulted in creating new ideas or seeking new ways so that they could reduce the problems and help the patients and colleagues. They mentioned that commitment to nursing profession and their feelings of responsibility, empathy and compassion resulted in helping human beings including patients and those concerned for their welfare. They stated high emotional sensitivity and altruistic behaviors helped them put themselves in the patient’s position and see the problems from the patients’ perspectives. Creative nurses pointed out that they could not be indifferent about pain and physical, psychological and economic problems of the patients in the hospital and they liked to help them. Finally they cited that they like to provide greater and better services to the patients, colleagues and community.

*“I feel the patients are one of my family members when I take care of them and I like to help them and do the best for them so that they feel easier” [nurse 6, 14 years work experience]*.

*“ Because I myself had experienced femur fracture previously, and I knew how much pain is there, so while taking care of these patients, the senses of empathy and sympathy helped me to spark this idea (a special kind of skin traction bed) in my mind”[nurse 7, 11 years work experience]*.

*“In my opinion, when patients or their families are confronted with a problem, I feel it as my own problem which should be solved. In fact I put myself in the patient’s position. The feelings of professional commitment and empathy do not allow me to be indifferent about the problems” [nurse 8, 14 years work experiences]*.

### 3.5 Spiritual Motivators

Participants integrated spirituality into creativity in the workplace and stated that spirituality enhances their personal well-being and creativity. Participants stated that they consider their creative efforts as reward in the hereafter, and in this way, they linked spirituality to creativity. They thought beyond themselves and believed that God supervise all the works they do. They stated that their creative efforts and attempts to meet the needs of patients are in the line with their religious beliefs and are listed as an action that pleases God.

*“I am very interested in nursing and I like what I do as a creative work to be considered as a divine reserve” [nurse 7, 11 years work experience]*.

*“I think if I do not seek a better work or a bigger service to patients, or offer new ideas, in fact, this will not enjoyable for me, and maybe it would have negative effects on my mental health and well-being”[nurse 4, a founder of nursing association]*.

## 4. Discussion

This study aimed to explore nurses’ experiences about the motivational factors that really motivate them to express creativity in clinical settings. Study findings revealed that nurses are creative and innovative individuals and a combination of multiple motivational factors such as intrinsic, extrinsic, achievement, altruistic and spiritual motivators trigger their creativity. There is no qualitative study about nurses’ creativity in Iran, and there are few foreign studies about this issue. Therefore comparing results of this study with other studies in nursing is limited. Our study findings indicate that different forms of motivational factors can trigger creativity among nurses. In this study, intrinsic motivators were the most prominent motivational factors that were mentioned by all creative nurses. Our study demonstrates that intrinsic motivators such as nurses’ interest and love to nursing tasks triggered their creativity expression in clinical settings. So we can say that intrinsic motivation is associated with higher levels of nurses’ creativity. According to Grant and Berry (2011) the empirical evidence linking intrinsic motivation to creativity is equivocal (George, 2007; Shalley et al., 2004). Some studies have shown that intrinsic motivation is associated with higher levels of creativity (e.g., Amabile, 1985; Amabile, Hill, Hennessey, & Tighe, 1994), whereas other researches have indicated weak or non- significant associations (e.g., Dewett, 2007; Perry-Smith, 2006; Shalley & Perry-Smith, 2001). Adams (2005) also noted that motivation is generally accepted as key to creative production, and the most important motivators are intrinsic passion and interest in the work itself. We can say that people will be most creative when they feel motivated primarily by their interest, satisfaction and challenge of the work itself (Amabile, 1998). According to Gilmartin (1999) organizational creativity also begins with creative people and stems from the ability to “do what you love and love what you do.” (Amabile, 1997). When this state of gratification is achieved, the potential for innovation and creativity is at a maximum. A love for one’s work stems from an internal motivation to engage in challenging, rewarding, and mind-expanding work. We also found that nurses’ creative efforts resulted in positive emotions such as satisfaction, happiness, high self-esteem, self-belief and so on. According to Grant and Berry (2011) from the standpoint of emotion theories, by fostering positive affect, intrinsic motivation enhances psychological engagement and builds energy for sustaining effort, increasing the amount of time that employees are willing and able to work on their tasks (e.g., Fredrickson, 1998). From the standpoint of self-determination theory, by fostering confidence and interest, intrinsic motivation encourages employees to persist with challenging, complex, unfamiliar tasks (Gagne´ & Deci, 2005) as well as to concentrate their attention more effectively on these tasks (e.g., Amabile, 1996).

The greatest potential for creative success is that nursing students and clinical nurses find their passion and interest and jump into it early in their work life. There is a concern that many nursing students graduate from universities as generalists and miss early opportunities to discover an area of their passion or interest in which to build deeper nursing knowledge. So nursing educational systems especially in Iran should provide greater focus on helping nursing students identify their areas of interest, areas in which they can achieve a state of flow that leads to the growth of their skills and confidence, the states under which creativity blossoms. Educational systems in Iran should set careful criteria to select more interested students in nursing and students should be interviewed as a part of entrance exam of the university.

We also found that nurses are motivated by several external motivating factors or external pressures such as confronting with clinical setting problems, work overload, time pressure, and so on. Our study findings demonstrated that intrinsic and extrinsic motivation can both impact nurses’ behavior, but in different ways. The findings indicated that external motivating factors can finally be internalized by creative nurses. On the other hand, nurses internalized the externally motivated behaviors. For instance, confronting with repeated problems in clinical settings which are known as external triggering agent can stimulate nurses’ sensitivity as a result of internal motivation or confront them with mental challenges. Creative nurses stated that when they were faced with problems, they could not be able to close their eyes and forget them; they react in a way to solve it. Clinical problems led to mental challenges and this led to thought about several diverse alternatives for solving problems and this in turn resulted in spark of novel and useful ideas. When their new ideas, products, services or procedures were used in hospitals, and they were encouraged and supported by managers and colleagues or they felt their new ideas were appropriate and useful, and resulting simplification in their own work or the works of other personnel, they felt satisfaction, happiness, self- confidence and other positive affect that encouraged them to do subsequent creative tasks. Amabile and Steven (2011) also noted that nearly all intrinsically-motivated tasks on the job have some extrinsic motivators attached. Research on various types of extrinsic motivation and the internalization of externally motivated behavior, however, is scarce. An exception is the study conducted by Grouzet, Vallerand, Thill and Provencher (2004) who found that environmental factors, such as success or failure, influenced perceptions of competence, autonomy and relatedness (psychological need factors). These factors, in turn, determined the extent to which self-determined motivation was exhibited. Study findings indicated that nurses internalize extrinsic motivation into intrinsic motivation. Ryan (1982) also noted that it is through the process of internalization that extrinsically motivated behaviors which are initially externally prompted can become increasingly internalized and result in greater self -regulation. Internalization means people’s transformation of external regulatory processes into internal regulatory processes (Deci & Ryan, 1996; Deci, Ryan, & Koestner, 1999).

The third theme of the study was achievement motivators. Study findings revealed that creative nurses worked hard and spent a lot of time and energy to do their best so that the patients receive the best care and the tasks are performed in simpler and easier way. Study findings also indicate that creative nurses do creative efforts because they like to be the first and the most excellent employee in the workplace. Marsh (2007) also proposed that achievement motivation was the reason why some people seem to be very keen to do well, while others seem to be reluctant to make an effort, and do mind whether they are successful or not. Conroy (2003) also highlighted that achievement motivation is working as a motivational factor for the effective functioning of creativity. Ghasemi, Rastegar, Ghorban Jahromi (2011) also found that among components of achievement motivation, hard working, purposefulness, and insistence had positive meaningful relation to entrepreneurship.

The forth theme was relational or altruistic motivators. Study findings demonstrated that deeply understanding of the clinical status and feeling the problems of clinical setting helped the nurses express creative efforts so that they could help other people such as patients, colleagues and so on. Potgieter (1999) noted that the creative solution or the creative idea is a goal which the individual tries to achieve through freeing the mind from the present conceptual system, and by exploring deeper or more comprehensive or clearer understanding of the situation. Study findings also revealed that the feelings of empathy and altruism helped nurses to express creativity. Altruism or selflessness is the principle or practice of concern for the welfare of others (Wikipedia). According to Amabile and Steven (2011) relational or altruistic motivation arises from the need to connect with and help others. The camaraderie that comes from collaborating with enjoyable colleagues can drive us in our work, and therefore can create the belief that our work is really valuable to a person, a group or the society at large. Liu, Cheng, Chao, Tseng (2012) also highlighted that altruism is a discretionary personal attitude in which behaviors are performed without expecting any further reward and are carried out mainly to benefit others. Since helping behavior can be considered as voluntary acts performed with the intention to provide some benefit to another person, altruistic intentions appear to be intrinsically motivated as a result of a consideration for the needs of others.

The fifth theme was spiritual motivators. Study findings revealed that participants linked spirituality into creativity in the workplace. They stated that spirituality enhances their personal well-being and creativity. We also found that nurses’ religious beliefs were important motivating factors to express creativity. Krishnakumar and Neck (2002) also noted that creativity isn’t created in vacuum, it needs a motive, spirituality can help people to develop their consciousness, increase their information, and it can relate creative power of human mind to God. Recently several researchers have also explored the relationship between spirituality and creativity in the workplace (Reave, 2005). A theoretical review of the literature indicates that spirituality might influence creativity in terms of motivation, social support and intention/receptivity (Daniel, 2007). Ziyaaddini and Zande Moghadam (2013) also found that there is a meaningful relation between spiritual life of employees and the rate of creativity in Kerman university executive systems. Accordingly, several dimensions of spirituality, when integrated into the workplace, can greatly enhance personal well-being and creativity, organizational harmony, and long-term business success. These are ultimate values, optimal human development, the art of transcendence, and spiritual psychologies, both ancient and modern (Butts, 1999). Spiritual intelligence is also considered by some people as an essential component of both personal and professional development, so managers can tap into team members’ spiritual intelligence to improve creativity, motivation and performance (Steve, 2013). And finally, Lane (2005) found that creativity and spirituality allow nurses to transform healing and change nursing care.

## 5. Conclusion

In this study, we tried to explore nurses’ experiences regarding motivational factors that trigger their creativity or expression of creativity in clinical settings. This qualitative study was performed for the first time in Iran and its results revealed that nurses are creative and innovative individuals and are able to overcome many complex health care challenges at their workplace. It was found that multiple motivational factors triggered nurses to express creativity, therefore creativity should be supported and fostered among them by nursing managers. In order to prosper creativity, health care organizations should provide a warm intellectual environment that gives nurses more recognition, prestige, and opportunity to participate. Nurse managers’ should increase motivation in nurses and promote creativity through being sensitive that in turn gives nurses the attention they want and treats them feeling of distinct individuals. When nurse leaders encourage nurses to express their ideas openly and accept divergent ideas, nurses will be motivated to be creative. Also by providing assistance for the development of new ideas and providing time for professional growth and development, encouraging nurses to interact with others outside their groups, recognizing the value of worthy ideas and promoting constructive competition inside and outside their groups, and showing confidence, they can motivates nurses to express their creativity.

Nurse managers need to know that creative behaviour is inherent in human nature and can be developed and taught purposefully. So, creativity training should be considered in nursing education curriculum and techniques of creativity training including, brain storming, brain writing, visualization, cueing, lateral thinking and divergent thinking should be used by nursing students and staff. Creativity is an empowering and enriching process, the expansion of creativity within health care systems will allow the evolution of professional nursing practice, improvement in care delivery, and organizational performance. And finally, creativity always begins with opening doors to better possibilities. According to Hughes (2006) Florence Nightingale introduced systematic handwritten records to the medical profession 150 years ago. It is time to take the next step.
